# A technique to generate synthetic CT from MRI for abdominal radiotherapy

**DOI:** 10.1002/acm2.12816

**Published:** 2020-02-11

**Authors:** Shu‐Hui Hsu, Pamela DuPre, Qi Peng, Wolfgang A. Tomé

**Affiliations:** ^1^ Department of Radiation Oncology Montefiore Medical Center Bronx NY USA; ^2^ Institute for Onco‐Physics Albert Einstein College of Medicine Bronx NY USA; ^3^ Gruss Magnetic Resonance Research Center Albert Einstein College of Medicine Bronx NY USA; ^4^ Department of Radiology Montefiore Medical Center Bronx NY USA

**Keywords:** MRI, radiotherapy, segmentation accuracy, synthetic CT

## Abstract

**Purpose:**

To investigate a method to classify tissues types for synthetic CT generation using MRI for treatment planning in abdominal radiotherapy.

**Methods:**

An institutional review board approved volunteer study was performed on a 3T MRI scanner. In‐phase, fat and water images were acquired for five volunteers with breath‐hold using an mDixon pulse sequence. A method to classify different tissue types for synthetic CT generation in the abdomen was developed. Three tissue clusters (fat, high‐density tissue, and spine/air/lungs) were generated using a fuzzy‐c means clustering algorithm. The third cluster was further segmented into three sub‐clusters that represented spine, air, and lungs. Therefore, five segments were automatically generated. To evaluate segmentation accuracy using the method, the five segments were manually contoured on MRI images as the ground truth, and the volume ratio, Dice coefficient, and Hausdorff distance metric were calculated. The dosimetric effect of segmentation accuracy was evaluated on simulated targets close to air, lungs, and spine using a two‐arc volumetric modulated arc therapy (VMAT) technique.

**Results:**

The volume ratio of auto‐segmentation to manual segmentation was 0.88–2.1 for the air segment and 0.72–1.13 for the remaining segments. The range of the Dice coefficient was 0.24–0.83, 0.84–0.93, 0.94–0.98, 0.93–0.96, and 0.76–0.79 for air, fat, lungs, high‐density tissue, and spine, respectively. The range of the mean Hausdorff distance was 3–29.1 mm, 0.5–1.3 mm, 0.4–1 mm, 0.7–1.6 mm, and 1.2–1.4 mm for air, fat, lungs, high‐density tissue, and spine, respectively. Despite worse segmentation accuracy in air and spine, the dosimetric effect was 0.2% ± 0.2%, with a maximum difference of 0.8% for all target locations.

**Conclusion:**

A method to generate synthetic CT in the abdomen was developed, and segmentation accuracy and its dosimetric effect were evaluated. Our results demonstrate the potential of using MRI alone for treatment planning in the abdomen.

## 1 INTRODUCTION

MR images have better soft tissue contrast than CT images; however, they are not often used alone in a radiation therapy workflow since the lack of electron density information precludes heterogeneity corrections — a part of the standard of care in treatment planning. The uncertainties introduced in the registration process of MR images with CT images affect the accuracy of treatment target delineation, and therefore affect treatment quality. To streamline the workflow by implementing MRI alone in the planning process, several methods have been proposed for generating synthetic CT from MRI in various body sites. These methods have been reviewed comprehensively by Edmund and Nyholm,^1^ Johnstone et al,^2^ and Owrangi et al.^3^

Previous studies have shown promising results in terms of synthetic CT quality, and demonstrated the potential of using MRI alone in the workflow for head^4^, ^5^, ^6^, ^7^, ^8^, ^9^, ^10^ and pelvis^11^, ^12^, ^13^, ^14^, ^15^ radiation therapy. Despite encouraging results in head and pelvis, few studies investigated the potential of using MRI alone in the abdomen. Bredfeldt et al^16^ presented a shape‐aided intensity‐based tissue classification method using a single imaging sequence to generate synthetic CT and showed its feasibility for liver stereotactic body radiotherapy (SBRT) dose calculations. Guerreiro et al^17^ used an automatic atlas‐based segmentation of tissue classes followed by a voxel‐based MRI intensity to Hounsfield unit (HU) conversion algorithm and showed its feasibility in photon and proton dose calculations for treating children with abdominal tumors. Liu et al^18^ used a three‐dimensional (3D) cycle‐consistent generative adversarial network (cycle GAN) to generate synthetic CT for liver proton therapy. The methods that require MR to be registered with CT as a training dataset may be difficult for abdomen due to geometric distortions and motion artifacts.

This study aims to investigate the feasibility of a synthetic CT method using a single MR imaging sequence and to evaluate the segmentation accuracy of the proposed method and its dosimetric effect for abdominal radiotherapy. To avoid potential geometric differences between CT and MR images in the abdomen due to geometric distortion of the MRI, internal organ motion, and setup uncertainty, we compared tissue segments generated from the proposed method with manually contoured segments on MR images. Bulk density assignment using population‐based HUs, shown to be appropriate by a previous study,^19^ was applied to these tissue segments to evaluate the dosimetric effect of segmentation accuracy.

## 2 METHODS AND MATERIALS

### 2.1 Image acquisition

Five volunteers participated in an institutional review board (IRB)‐approved prospective protocol (Number: 2017‐7745), and underwent abdominal MRI scans on a flat tabletop (Medibord Ltd, Nottingham, United Kingdom) customized for a 3T Philips Achieva MRI scanner (Philips Medical Systems, Cleveland, OH). One volunteer was scanned in feet first supine position while the others were scanned in head first supine position. No gadolinium contrast was used for the volunteer study. MR images were acquired using a 16 channel SENSE XL torso phased array coil. A 3D mDixon pulse sequence was employed and in‐phase, fat‐only, and water‐only images were generated online. Typical scanning parameters included: TR/TE1/TE2/flip angle = 4 ms/1.38 ms/2.6 ms/10º; FOV = 340/250/240 mm; matrix = 264 × 156; voxel size = 1.29 × 1.60 × 4 mm; pixel bandwidth = 1076 Hz per pixel. Volumetric MR images from this sequence were acquired in one single breath‐hold of about 16 sec at the end of inhalation. A respiratory belt was used to monitor breathing for the breath‐hold scans. A vendor‐provided online intensity correction and spatial distortion correction were utilized.

### 2.2 Tissue classification for synthetic CT generation

**Figure 1:**
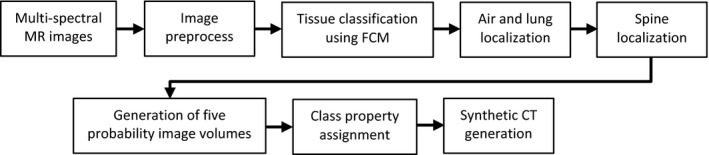
Tissue classification process for synthetic CT generation.

Our method utilized tissue characteristics present in MR images to classify tissues and assigned electron density to each tissue type. The method used a fuzzy c‐means (FCM) clustering algorithm with a spatial constraint, which was described by Hsu et al^20^ for synthetic CT generation in the head. The steps of this method are shown in Fig. 1. The input image volumes included in‐phase, fat and water MR images from the single pulse sequence to minimize possible classification errors due to image registration and internal organ motion in the abdomen between image acquisitions from different pulse sequences. The following sections describe the details in each step for synthetic CT generation in the abdomen.

#### 2.2.1 Image preprocessing

All MR images were corrected for residual intensity non‐uniformity using a commonly applied post‐processing bias‐field correction algorithm (N4itk)^21^ and implemented in a publicly available image analysis software environment (SLICER 4.6, surgical processing laboratory, Brigham and Women’s Hospital, Boston, MA). The bias field was estimated within the volume defined by the skin surface. The N4itk optimization parameters included: BSpline order of 3, BSpline grid resolutions of (1, 1, 1), a shrink factor of 4, maximum numbers of 50, 40, and 30 iterations at each of the three resolution levels, and a convergence threshold of 0.0001.

**Figure 2:**
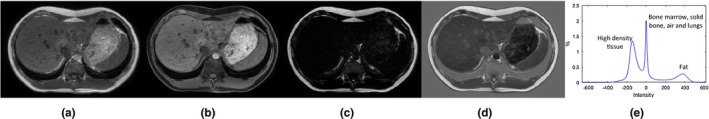
(a) In‐phase, (b) water, (c) fat, and (d) difference image (_d_MRI) volumes. (e) Intensity histogram inside body contours for the 3D volume of the _d_MRI. The histogram has three peaks, which include (1) high‐density tissue, (2) bone marrow, solid bone, air and lungs, and (3) fat.

A difference MR image (_d_MRI) volume was created by subtracting water images from fat images, which provided preferable tissue cluster distributions for classification in our method (Fig. 2). To reduce image noises on MR images, an edge‐preserving anisotropic diffusion filter was applied to all four MRI volumes (in‐phase, fat, water, and _d_MRI). A volume of interest (VOI) was segmented based on external contours on in‐phase MRI volume using Otsu’s thresholding method followed by morphological operations, such as hole filling, dilation, and erosion.

#### 2.2.2 Tissue classification using FCM

Probabilistic tissue classification was achieved via the FCM clustering algorithm on _d_MRI volume, and three probability image volumes were generated to represent three tissue clusters, which are consistent with the histogram shown in Fig. 2: (a) high‐density tissue (e.g., liver, heart, and muscle), (b) bone marrow, solid bone, air, and lungs, and (c) fat. The second tissue class mixed tissue types with a wide CT number range, which were further partitioned into lungs, air, and spine and described in next two sections.

#### 2.2.3 Air and lung localization

To identify the lung segment, the intensity thresholding was first applied on in‐phase images using the threshold value acquired from Otsu’s method in section 2.B.1 to segment low intensity voxels which mostly belonged to lungs, air, or solid bone (e.g. ribs). Given that the size of air pockets and solid bone was much smaller than the size of the lungs, small objects in the segment with an area smaller than 3500 voxels in each two‐dimensional (2D) image were removed. Therefore, only voxels in the lungs were preserved. Then a 3D region growing technique with seed points in the lung mask on in‐phase images was utilized, followed by morphological cleanup. To identify the air segment, morphological erosion was applied on the low intensity segment that excluded lungs, and followed by region growing and morphological cleanup.

#### 2.2.4 Spine localization

**Figure 3:**
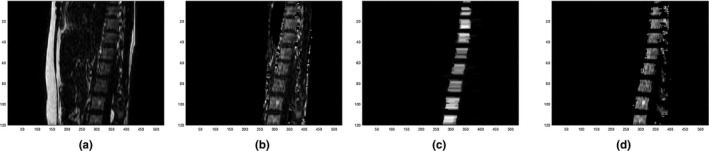
(a) A fat image after edge‐preserving filtering. (b) The fat image after removing fat, lungs, air, and non‐spine areas. (c) The fat image after performing the morphological opening and thresholding. (d) The fat image after performing region growing and removal of the spinal cord.

Our method primarily attempted to distinguish spine from other tissues, so ribs were ignored and classified as a mixture of high‐density tissue and fat. To localize the spine, a spine mask was first created based on its spatial location and dimension inside the body. Next, the spine mask was modified by removing voxels that also belonged to air, lungs, and fat, followed by a morphological opening to preserve bone marrow only. To keep the spinous processes, a solid bone mask was created simultaneously during the process for air and lung localization. Then, a 2D region growing technique with seed points in the bone marrow and solid bone masks was performed with intensity and distance constraints. The spinal cord was identified and removed from the spine mask based on its spatial location and cylindrical shape. Figure 3 shows the process for spine localization.

#### 2.2.5 Class property assignment to the probability image volumes for synthetic CT generation

**Figure 4:**
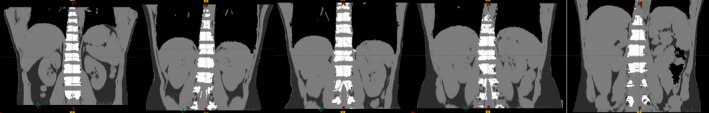
Synthetic CT in a coronal view for all five volunteers.

Because the second class was further classified into air, lungs, and spine, five probability image volumes were generated to represent air, lungs, spine, fat, and high‐density tissue. Population‐based HUs from our previous study were then applied to these five tissue classes: −1000 HUs for air, −708 HUs for lungs, −89 HUs for fat, 39 HUs for high‐density tissue, and 354 HUs for spine.^19^ The sum of probability‐weighted HUs in each voxels yielded the synthetic CT (Fig. 4).

### 2.3 Manual contouring on MR images

#### 2.3.1 Contours of five tissue segments

A ground truth contour set of fat, air, lungs, spine, and other high‐density tissue (i.e., residual tissue) was manually segmented on MR image volumes using a combination of the available drawing tools that are implemented in a commercial treatment planning system (Eclipse 13.7, Varian Medical Systems, Palo Alto, CA). First, an intensity thresholding tool was used to segment the fat and vertebral body on fat images. Air and lungs were contoured on in‐phase images. A 3D brush was then used to modify and clean‐up air and lung segments. A 2D adaptive brush on each axial slice was used to contour the spinous process and a rigid eraser was used to clean up the entire segment — slice by slice. The spine segment was post‐processed to remove stray pixels, fill in holes, and smooth the contour. The remaining tissue structure was created using Boolean operators to subtract other segments (i.e., fat, air, lungs, and spine) out of the body. The body contour was the same as the one created from auto‐segmentation in section 2.B. In addition, the spine contouring process was processed by another observer to evaluate inter‐observer variations, and to study the impact of its segmentation subjectivity.

#### 2.3.2 Contours of simulated targets

**Figure 5:**
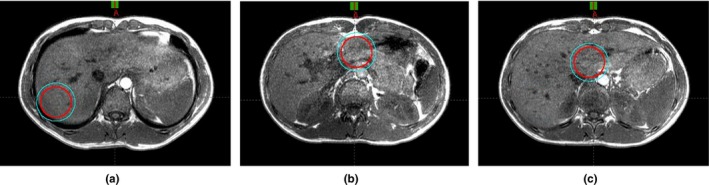
Simulated spherical targets, CTVs (red), and PTVs (cyan), adjacent to (a) lungs, (b) air pockets, and (c) spine displayed on in‐phase MR images. CTVs, clinical target volumes; PTVs, planning target volumes.

Three spherical clinical target volumes (CTVs) with a 2 cm radius were generated on MR images for all volunteer subjects. A 5 mm margin was applied symmetrically to the CTV to generate a planning target volume (PTV). The first target was centered at the upper abdomen adjacent to the lungs (CTV_lung_ and PTV_lung_), the second target was adjacent to the air pockets (CTV_air_ and PTV_air_), and the third target was approximately in the midline of the abdomen adjacent to the spine (CTV_spine_ and PTV_spine_) (Fig. 5).

### 2.4 Evaluation of segmentation accuracy

#### 2.4.1 Geometric comparison

Our method generates five probability maps, which indicate the probability of a given voxel to belong to a specific class. To compare them with manual segments, a defuzzification procedure was applied to convert this fuzzy partition to a crisp partition. Defuzzification involves a maximum membership conversion procedure in which a given the voxel *k* is assigned to the class *C_j_*, *j = 1,...N*, for which it has the highest membership probability (*u*).

Volume ratios, Dice coefficient, and Hausdorff distance metric were calculated using Velocity AI (Velocity 3.2.1, Varian Medical Systems, Palo Alto, CA), and compared between automatic segmentation and manual contouring. To evaluate overall segmentation accuracy, a weighted Dice coefficient ($\bar{S}$) was calculated by summing the Dice coefficient for each tissue segment *i* with the ratio of the segment volume (*V_i_*) to the total volume (*V_T_*):

(1) $$\bar{S}=\sum_{i=1}^{N}w_{i}S_{i}=\sum_{i=1}^{N}\frac{V_{i}}{V_{T}}S_{i}$$

where the sum of *w_i_* is equal to 1. The maximum $\bar{S}$ for the best segmentation is 1.

#### 2.4.2 Dosimetric effect of segmentation accuracy

The population‐based HUs (as described in section 2.B.5) were assigned to the five tissue segments that were generated from automatic segmentation after defuzzification and manual contouring. This resulted in a defuzzified MRCT (_d_MRCT) and reference MRCT (_r_MRCT). To quantify the dosimetric effect of segmentation accuracy, treatment planning employing hypo‐fractionated volumetric modulated arc therapy (VMAT) was performed on the _d_MRCT and _r_MRCT datasets in the Eclipse treatment planning system. Two partial arcs were utilized for planning on both PTV_lung_ and PTV_air_, whereas two full arcs were utilized for planning on PTV_spine_. The VMAT plan was first optimized on the _d_MRCT dataset following institutional constraints for target coverage and gradient falloff, and the dose was calculated using the analytical anisotropic algorithm (AAA) with a grid size of 1.25 mm. Then, the fluence was copied to _r_MRCT and the dose was recalculated. A total dose of 50 Gy in five fractions was utilized. Dose volume histogram (DVH) metrics for the doses to 97% (D97%) and 0.03 cm^3^ (D0.03 cm^3^) of the volume were extracted for the PTV and CTV and compared between _d_MRCT and _r_MRCT dose calculations.

## 3 RESULTS

### 3.1 Geometric comparison

**Figure 6:**
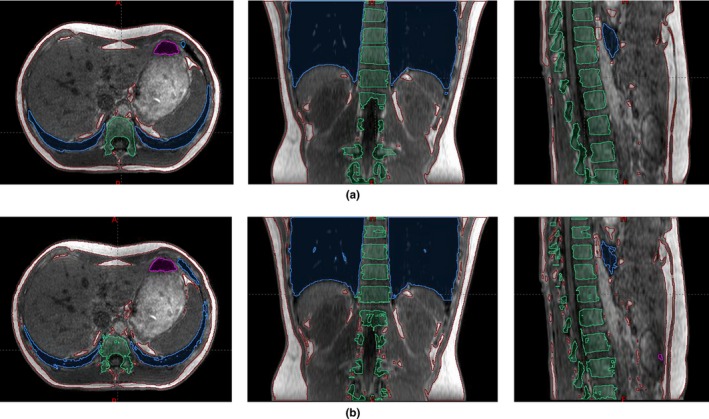
Air (magenta), lung (blue), fat (brown), and spine (green) contours using (a) manual and (b) auto‐segmentation for volunteer 2. The high‐density tissue segment includes the residual tissue (the contour is not shown).

Figure 6 shows the contours of the tissue segments generated manually and automatically. Table 1 shows the volume of manual segmentation, volume ratio of auto‐segmentation to manual segmentation, Dice coefficients, and Hausdorff distances in mm (mean ± one standard deviation) for the five volunteers. The volume ratio of auto‐segmentation to manual segmentation was 0.88–2.10 for the air segment while it was 0.72–1.13 for the remaining segments. The range of the Dice coefficient was 0.24–0.83 for air, 0.84–0.93 for fat, 0.94–0.98 for the lungs, 0.93–0.96 for high‐density tissue, and 0.76–0.79 for the spine. The range of the mean Hausdorff distance was 3–29.1 mm for air, 0.5–1.3 mm for fat, 0.4–1 mm for the lungs, 0.7–1.6 for high‐density tissue, and 1.2–1.4 mm for the spine. The segmentation accuracy was better for fat, lungs, and high‐density tissue, than it was for air. Although the accuracy of air was the worst, the overall segmentation accuracy using the weighted Dice coefficient ranged from 0.91 to 0.96 because the air volume was small in the abdomen and the majority of tissue types were fat, lungs, and high‐density tissue, which had better segmentation accuracy. Due to the laborious process of contouring the spine, spine contours were compared between two independent observers. The Dice coefficient for spine contours between two observers drawing on MR images was 0.95 ± 0.02 and the mean Hausdorff distance was smaller than 0.5 mm for all volunteers.

**Table 1:** The volume from manual segmentation, volume ratio of auto‐segmentation to manual segmentation, Dice coefficient, and Hausdorff distance (mm) for air, fat, lungs, high‐density tissue, and spine for five volunteers. The weighted Dice coefficient for all segments is also shown.

Tissue Segment	Volunteer Number				
	1	2	3	4	5
Volume from Manual Segmentation (cm^3^)					
Air	37	32	13	189	98
Fat	4884	2081	1587	3674	1468
Lungs	1236	1696	1559	1459	396
High‐density tissue	12077	6098	6111	6546	5794
Spine	415	230	223	241	227
Volume Ratio (Auto/Manual Segmentation)					
Air	1.28	1.28	2.10	0.88	1.45
Fat	0.86	0.91	0.72	0.78	0.81
Lungs	0.94	0.97	1.01	0.98	0.97
High‐density tissue	1.07	1.04	1.07	1.13	1.05
Spine	0.80	0.81	0.96	0.87	0.73
Dice Coefficient					
Air	0.238	0.830	0.596	0.823	0.587
Fat	0.920	0.935	0.844	0.880	0.891
Lungs	0.947	0.973	0.980	0.971	0.939
High‐density tissue	0.957	0.964	0.953	0.928	0.958
Spine	0.786	0.787	0.786	0.763	0.791
Weighted Dice	0.941	0.955	0.935	0.914	0.936
Hausdorff Distance in mm (Mean ± 1 SD.)					
Air	29.1 ± 34.9	4.1 ± 10.7	5.8 ± 11.1	3.0 ± 8.1	6.3 ± 10.3
Fat	0.5 ± 0.7	0.6 ± 1.1	0.8 ± 1.2	1.3 ± 1.9	0.5 ± 0.8
Lungs	1.0 ± 2.2	0.7 ± 1.8	0.4 ± 0.9	0.6 ± 1.3	0.8 ± 2.2
High‐density tissue	0.7 ± 1.3	0.8 ± 1.5	0.9 ± 1.4	1.6 ± 2.8	0.7 ± 1.3
Spine	1.4 ± 1.8	1.2 ± 1.4	1.3 ± 1.6	1.4 ± 1.9	1.3 ± 1.9

### 3.2 Dosimetric effect of segmentation accuracy

The impact of segmentation accuracy on dose calculations was evaluated for three different target locations that were adjacent to lungs, spine, and air (Table 2). Generally, the calculated dose to targets on _r_MRCT generated using manual segmentation was lower than the dose on _d_MRCT using auto‐segmentation. The difference for all target locations and volunteers was 0.2% ± 0.2%, with a maximum difference of 0.8%. No dosimetric difference was observed between target locations.

**Table 2:** Relative dose difference (%) in targets for the plans on _r_MRCT to the plans on _d_MRCT. Mean, one standard deviation, and range are shown.

Metrics	PTV D97%	PTV D0.03cm^3^	CTV D97%	CTV D0.03 cm^3^
Plan: PTV_lung_, CTV_lung_	−0.2% ± 0.2% [−0.5% 0%]	−0.2%±0.2% [−0.5% 0%]	−0.4% ± 0.2% [−0.8% −0.2%]	−0.2% ± 0.2% [−0.5% 0%]
Plan: PTV_spine_, CTV_spine_	−0.2% ± 0.1% [−0.3% 0%]	−0.2% ± 0.3% [−0.8% 0%]	−0.2% ± 0.1% [−0.4% 0%]	−0.2% ± 0.3% [−0.8% 0%]
Plan: PTV_air_, CTV_air_	−0.2% ± 0.2% [−0.4% 0%]	−0.3% ± 0.3% [−0.8% 0%]	−0.2% ± 0.3% [−0.7% 0.1%]	−0.3% ± 0.3% [−0.8% 0%]
All Target Plans	−0.2% ± 0.1% [−0.5% 0%]	−0.2% ± 0.3% [−0.8% 0%]	−0.3% ± 0.2% [−0.8% 0.1%]	−0.2% ± 0.3% [−0.8% 0%]

CTVs, clinical target volumes; dMRCT, defuzzified MRCT; rMRCT, reference MRCT. PTVs, planning target volumes.

## 4 DISCUSSION

In this study, the segmentation accuracy of our proposed method in generating synthetic CT was evaluated in the abdomen, and the weighted Dice coefficient was found to be between 0.914 and 0.955 for the five volunteer subjects. In addition, the impact of segmentation accuracy on dose calculations for target locations adjacent to lungs, spine, and air was evaluated and found to be within the clinically acceptable range, with a mean difference of 0.2% and a maximum difference of 0.8% in target coverage.

The segmentation accuracy was the worst for air, with a Dice coefficient of 0.24–0.83 and a Hausdorff distance of 3–29.1 mm. However, due to its small volume, the dosimetric effect was not critical. For spine segmentation, the Dice coefficient was 0.76–0.79 while the Hausdorff distance was 1.2–1.4 mm. Spine segmentation was the most challenging step in automatic segmentation on MR images because the signal in solid bone was really low and it could not be easily separated from air. In addition, the contrast of bone marrow was not sufficient to be completely separated from other tissues using the imaging sequence in our study. Misclassification was observed in the spine and the neighboring area; however, its dosimetric effect was not critical when using two full arcs on the target close to spine. Improving MR imaging contrast and spine localization accuracy would improve the quality of synthetic CT, and may be more critical when using synthetic CT for dose calculations in spine metastasis cases. Regarding the remaining tissue segments, the Dice coefficient was higher than 0.84 and the mean Hausdorff distance was less than 1.6 mm. Because the volume of these tissue types was much larger than air and spine, the dosimetric accuracy was found not to depend on target locations.

When manually contouring different tissue segments, there was a small uncertainty that resulted from the process in intensity thresholding and observer judgment. This may cause misclassification between high‐density tissue and other tissue segments (e.g., fat) because the high‐density tissue volume was acquired by subtracting the other four tissue segments from whole body volume. This may explain why the volume ratio was larger than 1 for high‐density tissue and smaller than 1 for fat. The inter‐observer variation was within 5% based on the Dice coefficient on the spine segment. The uncertainties in the process of manual contouring would have a minimal effect on the evaluation of segmentation accuracy on dosimetry in our study.

The segmentation accuracy using the proposed method resulted in a mean dose difference of 0.2% for target coverage when using VMAT in the abdomen. Using population‐based HUs on five tissue segments resulted in a mean difference of 0.1% for target coverage according to our previous study.^19^ The dosimetric uncertainty caused by MRI geometric distortion only was ~0.5% when the distortion was 3 mm.^22^ Therefore, the combined uncertainty of distortion, segmentation accuracy, and population‐HU assignment is estimated to be ~0.6%. This result indicates that the dosimetric accuracy is clinically acceptable and demonstrates the potential of using MRI alone for treatment planning in the abdomen.

The proposed method only used one single MR imaging sequence, which minimizes the uncertainty of movement between imaging sequences and therefore improves the quality of synthetic CT. In addition, it does not require MRI‐CT pairs as a training dataset, and the uncertainty from mismatch between MRI‐CT due to motion and distortion is eliminated. Guerreiro et al^17^ reported a dose difference of 0.5% for ITV coverage with VMAT when compared with true CT using an automatic atlas‐based segmentation with a triple‐model technique for MRI intensity to HU value conversion for pediatric patients under general anesthesia. Due to less motion artifacts in the training data under general anesthesia, their results and our results were within the same order of magnitude. Our method is similar to the method presented by Bredfeldt et al.^16^ The main difference is that the authors applied a vertebral body shape model which separated bone from air but ignored spinous processes, while our method used the intensity thresholding, areas, and spatial locations of the spine and kept the spinous processes. However, the dosimetric effect of spinous processes was negligible because their reported dosimetric accuracy was ~0.3% although their comparison was made on synthetic CT vs. true CT.

To evaluate segmentation accuracy, the probability maps generated from the proposed method were defuzzified so the information of intensity variations in the voxels disappeared. Future work will optimize the parameters in the clustering algorithm to retain tissue characteristics within each voxel, and dosimetric accuracy will be evaluated by comparing synthetic CT with true CT in a large patient cohort. In addition, a comparison with other available methods is warranted.

## 5 CONCLUSION

A method to classify different tissue types for synthetic CT generation in the abdomen was developed, and segmentation accuracy and its dosimetric effect were evaluated. The segmentation accuracy in the air segment was worse than the other tissue segments (i.e., fat, lungs, high‐density tissue, and spine). The Dice coefficient was higher than 0.76 and the mean Hausdorff distance was less than 1.6 mm for all tissue segments except for air. The mean dose difference for target locations close to air, lungs, and spine was 0.2%, with a maximum difference of 0.8%. Our results demonstrate the potential of using MRI alone for treatment planning in the abdomen.

## CONFLICT OF INTEREST

The authors have no conflicts of interest to disclose.
